# Phosphorylation of Kapok Fiber with Phytic Acid for Enhanced Flame Retardancy

**DOI:** 10.3390/ijms232314950

**Published:** 2022-11-29

**Authors:** Xin-Lin Jiang, Ren-Cheng Tang

**Affiliations:** 1 College of Textile and Clothing Engineering, North Compus, Soochow University, Suzhou 215021, China; 2 Jiangsu Engineering Research Center of Textile Dyeing and Printing for Energy Conservation, Discharge Reduction and Cleaner Production (ERC), North Compus, Soochow University, Suzhou 215123, China; 3 China National Textile and Apparel Council Key Laboratory of Natural Dyes, North Compus, Soochow University, Suzhou 215123, China

**Keywords:** kapok fiber, flame retardant, phosphorylation, phytic acid, thermal stability

## Abstract

Kapok fiber (KF), with the characteristics of a natural hollow structure, light weight, and low density, can be used as acoustic and thermal insulation, buoyancy, adsorption, filling, and composite material. The flame-retardant treatment can expand the functionality and application of KF. In this work, the phosphorylation of KF using phytic acid (PA) in the presence of urea at a high temperature was used to enhance its flame retardancy. The phosphorylation reaction conditions were discussed, and the surface topography, thermal degradation, heat release, and combustion properties of phosphorylated KF were studied. The Fourier transform infrared spectroscopy and ^31^P solid-state nuclear magnetic resonance spectroscopy analyses confirmed the grafting of PA on cellulose by the formation of phosphate ester bonds. Due to the covalent binding of PA, phosphorylated KF exhibited good washing durability. The surface topography, Raman spectroscopy, thermogravimetric (TG), and microcalorimetry analyses revealed the excellent charring ability of phosphorylated KF. In the TG test in nitrogen, the char residue increased to 42.6% of phosphorylated KF from 8.3% of raw KF at 700 °C. In the vertical combustion, raw KF sheet was almost completely burned out within 30 s, while phosphorylated KF was very difficult to catch fire. In the microcalorimetry analysis, the heat release capacity and total heat release of phosphorylated KF decreased to 67 J/g∙K and 3.9 kJ/g, respectively from 237 J/g∙K and 18.1 kJ/g of raw KF. This work suggests that phosphorylated KF is an excellent flame-retardant material.

## 1. Introduction

Kapok fiber (KF) is a natural single-celled fruit fiber that is extremely high hollow (up to 80–90%) and high lignification [1]. This fiber has a hollow tube structure with a wall thickness of 0.8–1.0 μm and a diameter of 8–10 μm [2], which makes it very light in weight and low in density. The unique structure and properties of KF have attracted considerable attention [3]. In recent years, KF has been mainly used as acoustic and thermal insulation [4,5], buoyancy [6], adsorption [1], filling [7,8], and composite materials [9].

The flame-retardant modification can improve the functionalities of KF and broaden the applications of KF. When KF is used as a filling material for bedding, waddings, pillows, quilts, cushions, soft toys, upholsteries, and car interiors, as well as composite materials, the flame-retardant treatment is necessary [10,11]. Although a great amount of fruitful research has been done on the most widely used cotton textiles, the flame-retardant treatment of KF has rarely been reported. In the past, cotton fabrics were commercially treated with two flame retardants, *N*-methyloldimethylphosphonopropionamide, and tetrakis-(hydroxymethy1)-phosphonium hydroxide-ammonia, by a pad-dry-cure technology for enhanced flame retardancy [12,13]. However, such an approach is seldom applied in the flame-retardant treatment of staple fibers. In addition, flame retardants containing methylol groups have the issue of formaldehyde release [14]. Therefore, novel and eco-friendly treatments should be pursued to improve the flame retardancy of KF loose fibers. In recent years, bio-based flame retardants have come into the limelight, as they are generally harmless to the environment and human health, among which biogenic phytic acid (PA) is the most representative [15,16].

Phytic acid (PA), also known as inositol hexaphosphate, is extremely high in phosphorus due to the presence of six phosphate groups, and it is found in high concentrations in cereals, oilseeds, and pulses [17]. Due to high phosphorus content, PA and PA salt (phytate) have been used to improve the flame retardancy of composite materials and textile fibers [18,19]. As PA is used as a flame retardant, it stimulates the degradation of cellulose below 300 °C, catalyzes the dehydration reaction, and increases the carbonization rate [19,20]. Thus, fibrous fabrics containing PA in flame retardants form a protective layer that inhibits the further combustion of fabrics [21].

In the textile industry chain, the pretreatment, dyeing, and finishing of loose fibers are usually carried out using batch machines and seldom using continuous processing machines. The classical flame retardants for cellulose fabrics are commonly applied using the pad-dry-cure technology, which is a continuous process. In light of the processing features of loose fibers, in the present study, the flame retardancy of KF loose fibers was enhanced by phosphorylation reaction with PA using an immersion-dry-high temperature reaction approach. It is hypothesized that in actual production, KF loose fibers are first immersed in flame retardants, then centrifugally dewatered, and finally treated in a drying machine for drying and phosphorylation reaction. Compared with the phosphorylation by conventional phosphoric acid and phosphate salts produced from phosphate rock, the phosphorylation by PA has the advantage of being environmentally friendly and can meet the requirements of green chemistry. In order to increase the extent of phosphorylation, the pretreatment of KF loose fibers was carried out using caustic soda with the additional aim of removing impurities [22,23,24], and urea was used as a swelling agent of cellulose and a catalytic agent of phosphorylation reaction [25,26]. In this work, the conditions of phosphorylation reaction of KF loose fibers were first discussed, and the resulting KF samples were characterized by Fourier transform infrared (FT-IR) spectroscopy, ^31^P solid-state nuclear magnetic resonance (NMR) spectroscopy, and inductively coupled plasma-atomic emission spectrometry (ICP-AES). Later, the thermal degradation and heat release properties of phosphorylated KF were investigated by thermogravimetric (TG)and pyrolysis combustion flow calorimetry (PCFC) analyses, respectively, the combustion performance of phosphorylated KF sheet samples was tested by a vertical combustion approach, and the surface topographies of raw KF and phosphorylated KF as well as their burned residues were studied by scanning electron microscope (SEM). Moreover, the charring ability and flame retardancy mechanism of phosphorylated KF were elucidated.

## 2. Results and Discussion

### 2.1. Conditions of Phosphorylation

**Figure 1:**
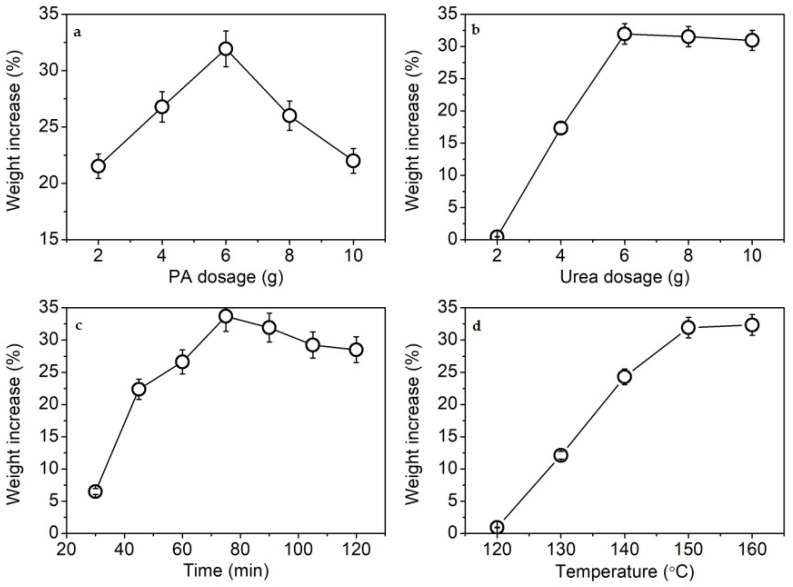
Weight increase percentage of KF at various PA dosages (**a**), urea dosages (**b**), reaction times (**c**), and reaction temperatures (**d**).

In a preliminary test, KF showed an increase in weight after phosphorylation. Therefore, the weight increase percentage was used to express the phosphorylation extent of KF in order to simplify the study on phosphorylation conditions. Figure 1a shows that the weight increase of KF was highest when 6 g of PA dosage was used. An excess of PA lowered the weight increase of KF. A similar phenomenon was also found in the phosphorylation of soft-wood kraft fiber with phosphoric acid [27]. The reduction in the weight increase percentage at high dosages of phosphorylating agents is caused by the degradation of cellulose [25,27].

In the phosphorylation reaction of cellulose, urea is often employed as a swelling agent of cellulose, a catalytic agent of reaction, and a neutralization agent of acid for improving the extent of phosphorylation and preventing the degradation of cellulose [25,26,27,28,29,30]. Figure 1b shows the distinct facilitating effect of urea on the phosphorylation reaction. At 6 g of urea, the highest extent of phosphorylation was achieved. The catalytic action of urea was further confirmed by the subsequent FT-IR analysis.

The time dependence of the phosphorylation reaction is shown in Figure 1c. The more efficient phosphorylation of cellulose occurred when the reaction time was 75–90 min. However, the longer time led to a slight decrease in the weight increase percentage of KF, likely due to the degradation of cellulose [25].

In a dry state with the presence of urea, the highly efficient phosphorylation of cellulose and starch occurs near or above the melting point of urea (132–135 °C) [31,32,33]. Urea and its degraded product (ammonia) can facilitate the swelling of cellulose and starch [32], thereby increasing the phosphorylation extent. Figure 1d shows that the weight increase percentage of KF increased with rising temperature between 120 and 150 °C due to the enhanced swelling of KF. The optimum phosphorylation temperature was 150 °C.

**Figure 2:**
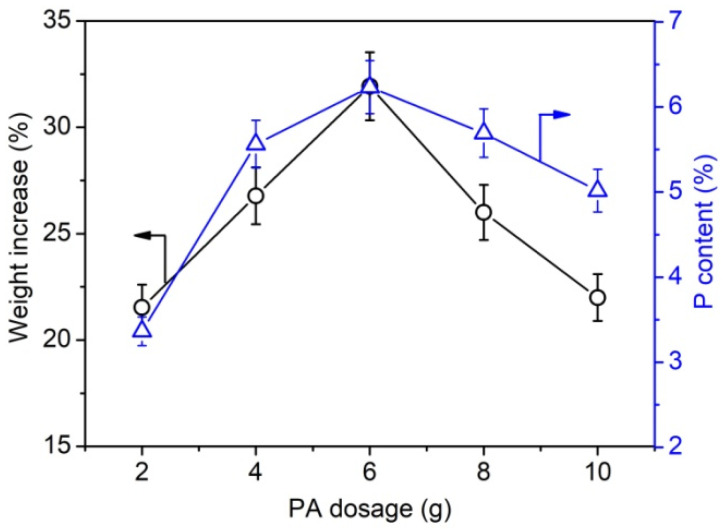
Weight increase percentage and phosphorus content of phosphorylated KF samples prepared using various dosages of PA (PA 2–6 g, urea 6 g, 150 °C, and 90 min).

**Figure 3:**
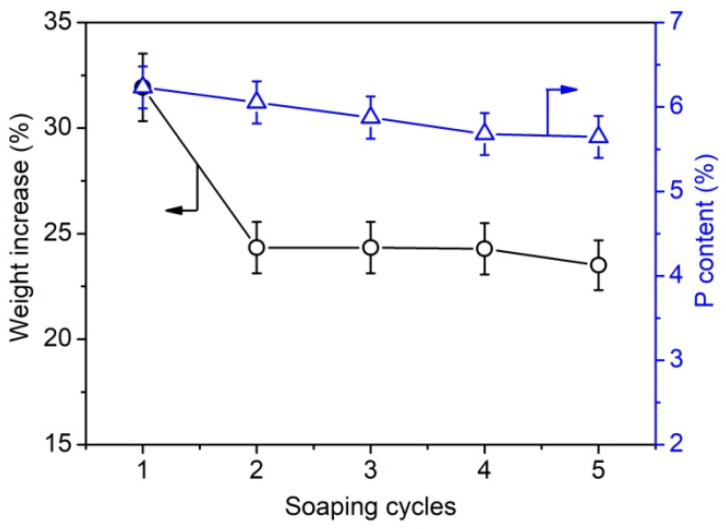
Weight increase percentage and phosphorus content of phosphorylated KF sample prepared using 6 g PA and 6 g urea at 150 °C for 90 min after different cycles of soaping.

Furthermore, the weight gain and phosphorus content of phosphorylated KF samples prepared using various dosages of PA and 6 g of urea at 150 °C for 90 min were determined. Figure 2 shows that the phosphorus content of phosphorylated KF also increased with increasing PA dosage and decreased after PA dosage exceeded 6 g. This trend is consistent with the change in the weight increase percentage of phosphorylated KF, indicating that the weight increase percentage can characterize the phosphorylation extent of KF. When the dosage of PA was 2, 4, and 6 g, the phosphorus content of phosphorylated KF was 3.36%, 5.56%, and 6.23%, respectively. As depicted in Figure 3, after two cycles of soaping, the weight increase percentage of phosphorylated KF showed a little change, whereas the phosphorus content decreased gradually with increasing cycles of soaping but still retained a high value (5.65%) after five cycles of soaping. The soaping test demonstrates the good washing resistance of phosphorylated KF, which is attributable to the formation of covalent bonding between KF and PA.

### 2.2. FT-IR Spectra of Phosphorylated KF

**Figure 4:**
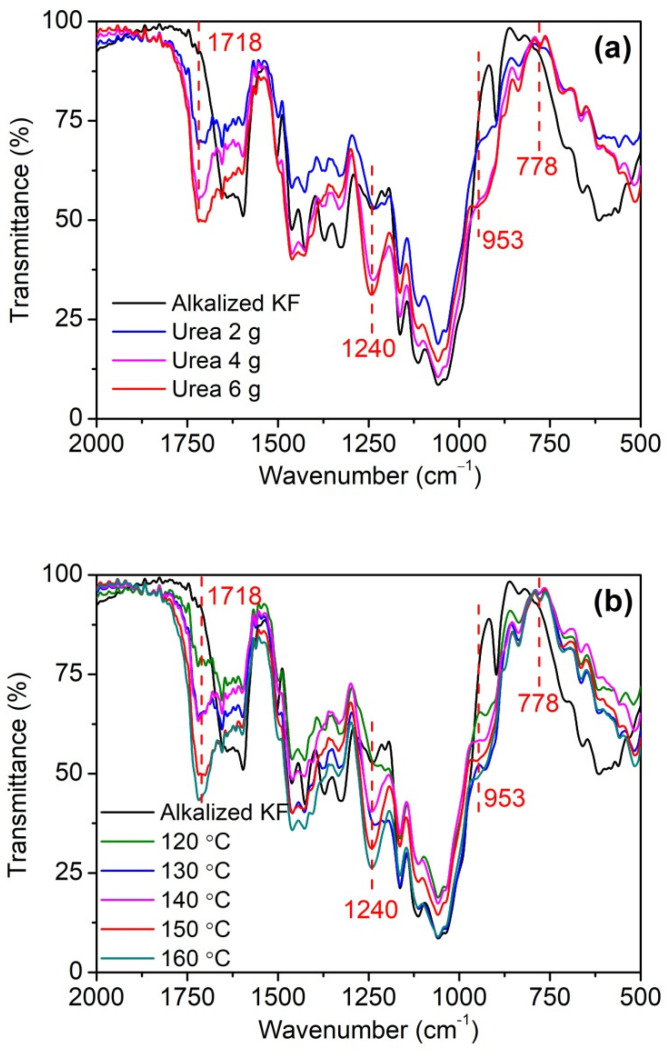
FT-IR spectra of alkalized KF and phosphorylated KF samples obtained at various urea dosages (PA 6 g, urea 2, 4, and 6 g, 150 °C, and 90 min) (**a**) and various temperatures (PA 6 g, urea 6 g, 120, 130, 140, 150, and 160 °C, and 90 min) (**b**).

**Figure 5:**
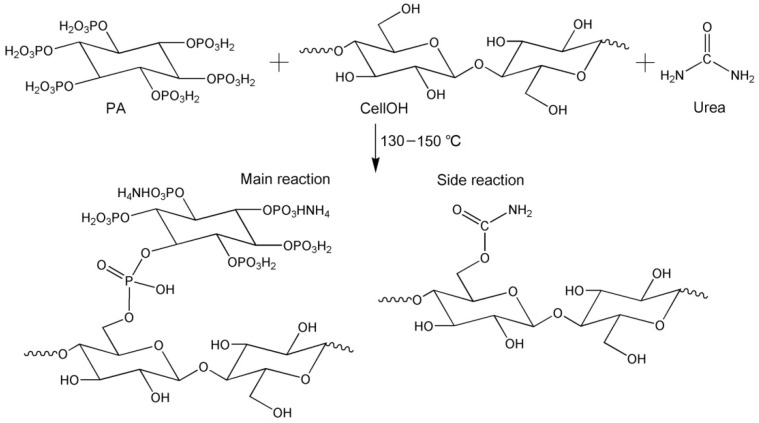
Schematic diagram of the phosphorylation of KF with PA in the presence of urea.

As can be seen in Figure 4, phosphorylated KF displayed three new peaks at 1718, 953, and 778 cm^−1^ compared with alkali-treated KF. The peak at 1718 cm^−1^ is the stretching vibration of C=O, which may be caused by the reaction product of urea and cellulose [34]. During the heating, urea decomposes to form isocyanic acid, which reacts with the hydroxyl group of cellulose to form a carbamate group [34]. In addition, the intensity of this peak gradually increased with increasing urea dosage (Figure 4a), indicating that urea participates in the reaction during the phosphorylation of cellulose. Furthermore, this peak also showed an increasing intensity with increasing temperature (Figure 4b), which is related to the increased degree of esterification of cellulose [31]. The peaks at 778 and 953 cm^−1^ correspond to P-O-C and P-OH, respectively [35,36]. Compared with alkalized KF, phosphorylated KF displayed an obvious peak at 1240 cm^−1^ with increasing intensity as urea dosage and temperature increased, which corresponded to P=O [37,38]. These peaks mentioned above demonstrate the linkage of the phosphate groups of PA onto cellulose. The schematic diagram of the phosphorylation of KF with PA in the presence of urea is presented in Figure 5.

### 2.3. ^31^P-NMRSpectrum of Phosphorylated KF

**Figure 6:**
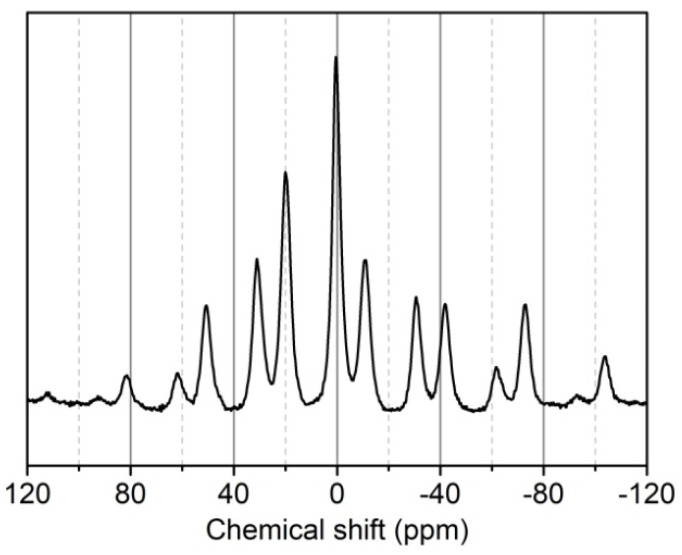
^13^P-NMRspectrum of phosphorylated KF sample prepared using 6 g PA and 6 g urea at 150 °C for 90 min.

In the ^31^P-NMR spectrum of phosphorylated KF (Figure 6), the main ^31^P signal appeared at 0.459 ppm, which is typical for phosphate ester bound to cellulose [38]. The presence of this signal demonstrates the grafting of PA onto cellulose. The signal at 20.033 ppm indicates the presence of phosphonates [39]. The signal at −11.101 ppm is assigned to polyphosphates which arise from the partial condensation of phosphate groups of PA present in KF during the heating step [28,39,40]. Moreover, other peaks at high chemical shift positions are rotational sidebands due to powder anisotropy [37,41,42].

### 2.4. Thermal Degradation Performance of Phosphorylated KF

**Figure 7:**
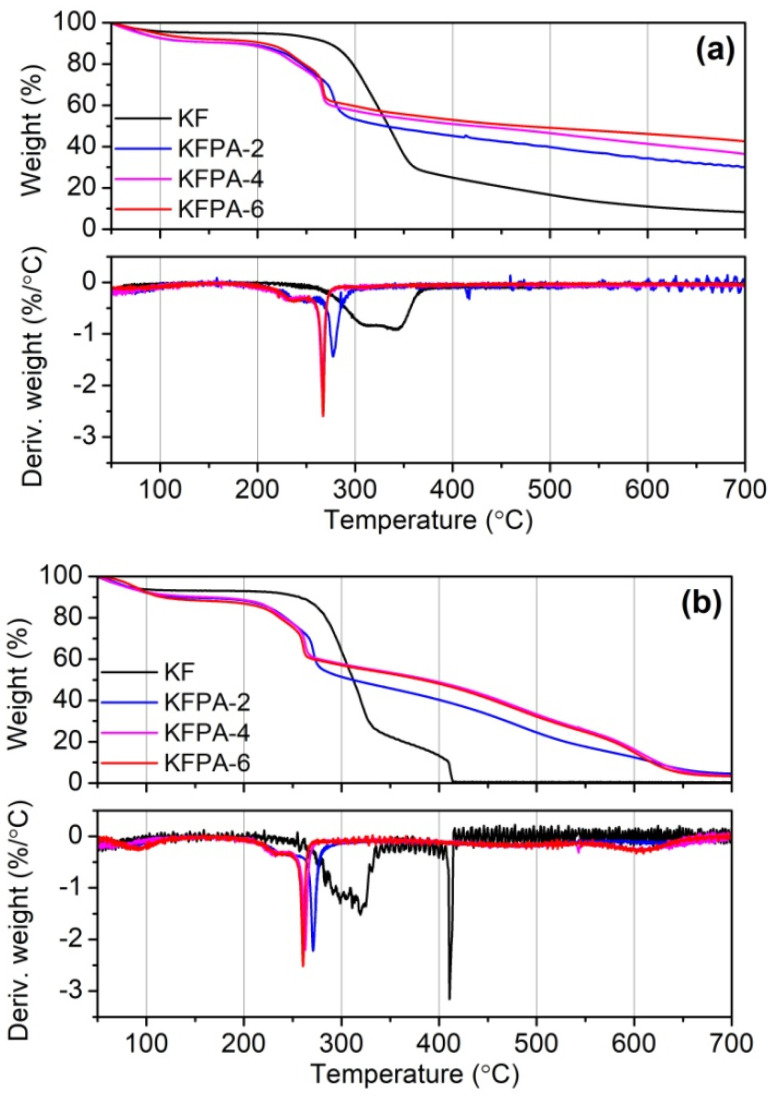
TG and DTG curves of KF and phosphorylated KF samples prepared at various PA dosages (PA 2, 4, and 6 g, 150 °C, and 90 min) in nitrogen (**a**) and air (**b**).

The TG and DTG (differential thermogravimetric) curves for raw and phosphorylated KF samples in nitrogen and air are shown in Figure 7, and the partial TG data are summarized in Table 1. In the nitrogen atmosphere, raw KF underwent two main weight loss stages. The first stage occurred in the temperature range of 280–360 °C with a maximum degradation rate temperature of 344.6 °C, which is due to the depolymerization of cellulose to form levoglucose and further decomposition [43]. The second stage occurred above 360 °C, during which the above degraded products further crack into low weight products and char. Compared with raw KF, phosphylated KF samples displayed lower onset degradation temperature (*T*_o_), end degradation temperature (*T*_e_), and temperature at the maximum degradation rate (*T*_max_), as well as much higher residual weights at *T*_max_, *T*_e_, 500 °C, and 700 °C, as shown in Table 1. Moreover, these residual weights increased with increasing PA dosages used in the phosphorylation reaction. These results indicate that phosphorylated KF samples degrade rapidly at lower temperatures, and their pyrolysis products have better thermal stability. These phenomena are associated with the fact that PA grafted onto cellulose is thermally degraded to produce phosphoric acid, which catalyzes the hydrolysis and charring of cellulose [19,20,44].

In air, the degraded products of all KF samples underwent further cracking and oxidation after the first degradation stage. Similarly, phosphylated KF samples displayed higher residual weights at high temperatures than KF. From the TG analysis, it can be deduced that phosphorylated KF samples possess good charring ability during high-temperature decomposition.

### 2.5. Heat Release Performanceof the Degraded Products of Phosphorylated KF

**Figure 8:**
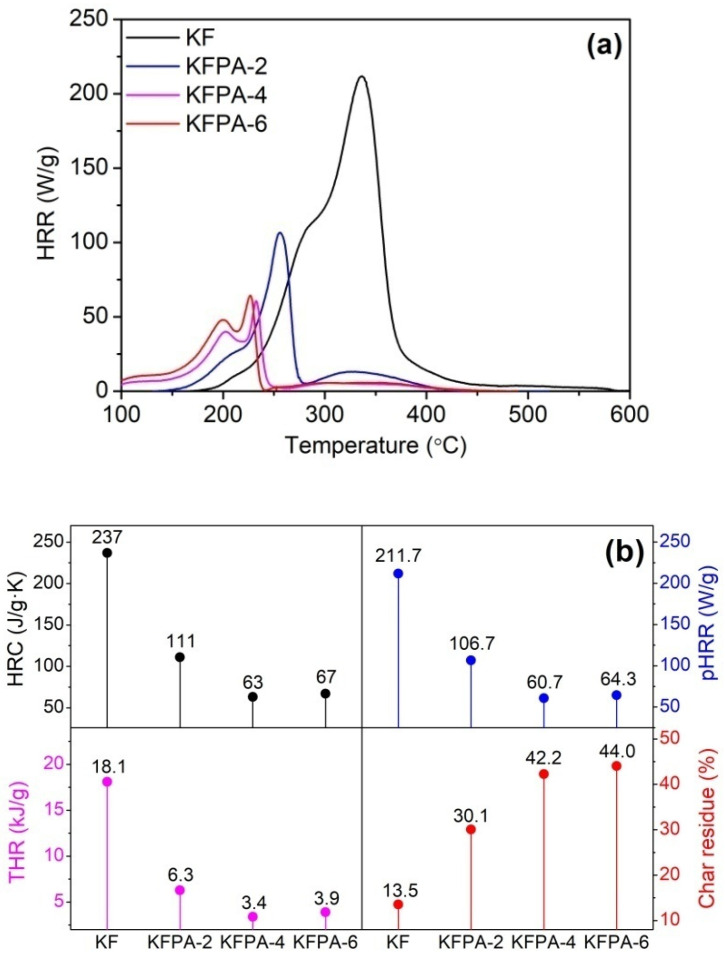
Heat release rate (HRR) curves (**a**) and PCFC parameters (**b**) of KF and phosphorylated KF samples prepared at various PA dosages (PA 2, 4, and 6 g, 150 °C, and 90 min).

In the PCFC technique, phosphorylated KF samples were first subjected to thermal degradation, and then, the fuel gases released from the degraded products were mixed with oxygen, and their flammability parameters were measured. As shown in Figure 8, phosphorylated KF samples showed much lower temperatures at the maximum heat release rate (*T*_max_), peak heat release rate (pHRR), heat release capacity (HRC), and total heat release (THR) of phosphorylated KF samples than raw KF. Moreover, phosphorylated KF samples showed much more char residues, which is consistent with the TG data. In the PCFC analysis, the lower *T*_max_ and more char residues of phosphorylated KF samples are also attributable to the hydrolysis and charring of cellulose caused by the acidic degraded products of PA. Additionally, Figure 8 shows that the phosphorylated KF samples prepared using 4 and 6 g of PA had lower pHRR, HRC, and THR values, and higher residual char quantities than the sample obtained using 2 g of PA. The PCFC analysis reveals that phosphorylated KF samples possess good charring ability in the process of thermal decomposition, and their degraded products exhibit low heat release behavior. Therefore, phosphorylated KF samples are good flame-resistant materials.

### 2.6. Flammability of Phosphorylated KF

**Figure 9:**
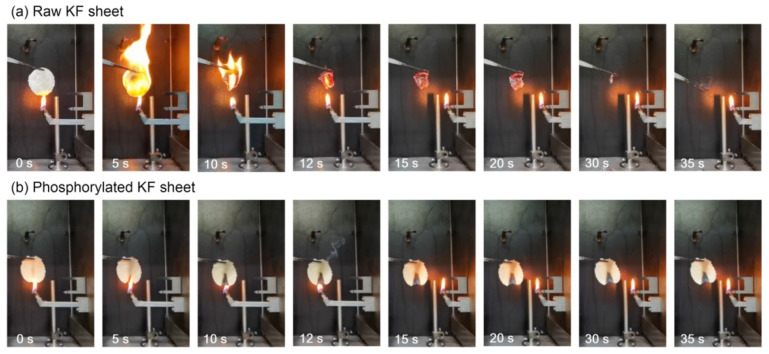
Flammability test of raw KF sheet (**a**) and phosphylated KF sheet obtained using 6 g PA (**b**).

To facilitate the evaluation of the flame retardancy of phosphorylated KF, sheet samples were used in the vertical combustion experiment. KF sheets were ignited for 12 s, and then the fire source was taken away. Figure 9 shows the images of the burning of raw and phosphylated KF sheets at different time intervals. Raw KF sheet easily caught fire, and rapidly burned once ignited. It was almost completely burned out in 30 s, eventually yielding a little bit of ash. However, phosphylated KF sheet could hardly be ignited, and during the ignition, it only had smoldering and no flaming combustion. After the completion of the ignition, the smoldering also stopped. At last, the intact char layers were left in the burned area. These results indicate that phosphorylated KF exhibits excellent charring ability and fire resistance.

### 2.7. Surface Topography of Unburned and Burned Phosphorylated KFs

**Figure 10:**
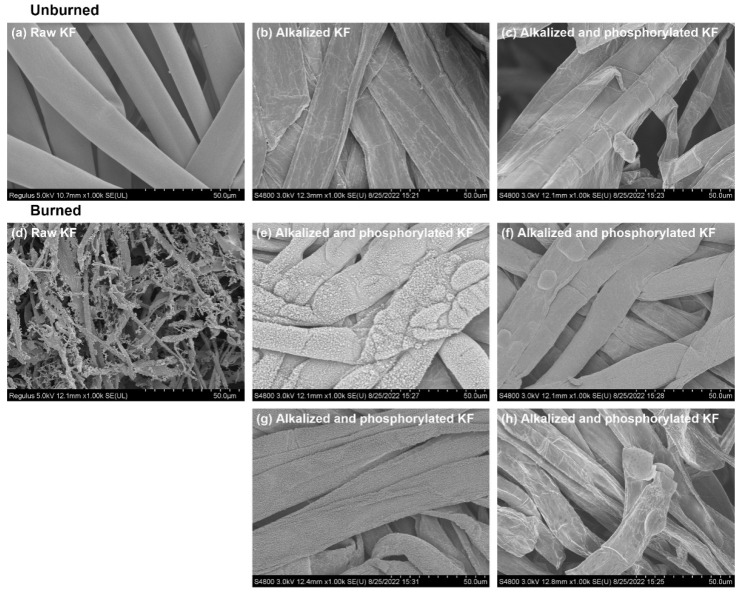
SEM micrographs of raw, alkalized, and phosphorylated KF sheets before and after combustion: Sheet samples: (**a**,**d**–**g**) and loose fiber samples: (**b**,**c**,**h**); (**e**,**f**) were collected from two sides of residual char in the middle of burned KF sheet; g was collected from the upper residual char of burned KF sheet; phosphorylated KF sample was obtained using 6 g PA.

As shown in Figure 10, contrary to the clean surface of raw KF (Figure 10a), the surface of alkalized and phosphorylated KF (Figure 10b,c) was squished and rough. The flattening shape of KF is caused by alkalization, which is consistent with the previous report. This result is consistent with the previous report in which alkalization made the air entrapment inside KF disappear, and alkalized KF looked like a flat ribbon [45]. Other studies showed that flat regenerated cellulose fiber could greatly improve the tear strength and quality of paper sheets [46,47]. Therefore, in addition to flame retardancy, the flat surface topography of phosphorylated KF may have potential application in reinforcing composite materials.

Burned KF sheets prepared using KF fibers with and without phosphorylation were collected from the test of Figure 9 for the SEM analysis (Figure 10d–g). Additionally, a staple fiber was ignited by a cigarette lighter, and its residue was analyzed by SEM (Figure 10h). As mentioned in the previous section, raw KF sheet yielded a little bit of ash after being combusted, which was fluffy and brittle. Correspondingly, the complete disintegration of raw KF was found in the SEM picture of its residual ash (Figure 10d). However, a distinctly different topography was observed for burned phosphorylated KF. After combustion, phosphorylated KF sample held the fiber shape, as shown in the SEM pictures of its residues collected from different areas of burned sheet (Figure 10e–g) and burned staple fiber (Figure 10h). This demonstrates the presence of firm char layers.

### 2.8. Raman Spectra of Char Residues of Raw and Phosphorylated KFs

**Figure 11:**
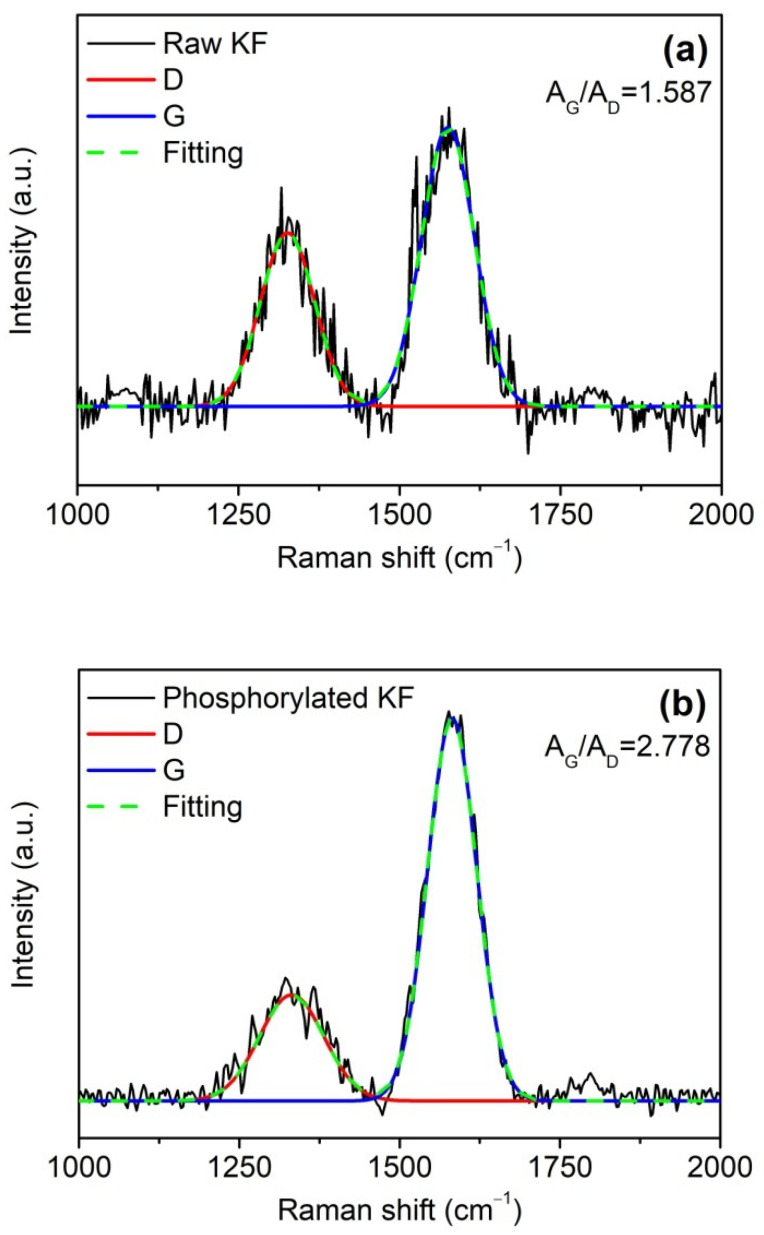
Ramanspectra of the residual char of raw KF (**a**) and phosphorylated KF (**b**) after combustion; phosphorylated KF sample was obtained using 6 g PA.

In the combustion process, the graphitization of polymers usually occurs. Therefore, Raman spectroscopy was used to evaluate the graphitization extent of the char residues of raw and phosphorylated KFs in order to provide further evidence for the enhanced charring ability of phosphorylated KF. In Figure 11, the Raman spectra of raw and phosphorylated KF showed two peaks at 1335 cm^−l^ (D-band) and 1580 cm^−l^ (G-band), which are assigned to the disordered or amorphous carbon and graphitized carbon, respectively [18,37]. The integrated peak area ratio of G to D bands (A_G_/A_D_) can characterize the graphitization extent of carbonaceous materials. Phosphorylated KF residue exhibited a graphitization extent than raw KF residue. This result indicates that phosphorylated KF yields more graphitic char during the combustion, which is very helpful in improving the flame retardancy of KF.

The surface topography, TG, PCFC, and Raman spectroscopy analyses demonstrate that phosphorylated KF exhibits excellent charring ability. The PCFC reveals that the degraded products of phosphorylated KF exhibit low heat release capability. The firm char layers formed during combustion can inhibit the emission of heat energy and flammable gases as well as the contact of oxygen with materials, thus having a good flame-retardant effect [48]. In other words, the flame-retardant mechanism of phosphorylated KF takes effect in the condensed phase.

## 3. Materials and Methods

### 3.1. Materials

Raw KF produced in Nanning City, Guangxi Province, China, was purchased from an online shop. Phytic acid (70%wt) was the product of Chengdu Boruite Chemical Technology Co., Ltd., Chengdu, China. Sodium hydroxide and urea were bought from Shanghai Tita Scientific Co., Ltd., Shanghai, China. A nonionic surfactant (fatty alcohol polyoxyethylene ether) used as a wetting agent was obtained from Sinopharm Chemical Reagent Co., Ltd., Shanghai, China. Soap chip originally applied for the washing color fastness test was purchased from Shanghai Textile Industry Technical Supervision Institute, Shanghai, China, and used as a detergent for the washing of phosphorylated KF samples.

### 3.2. Phosphorylation of KF

Before phosphorylation, KF was treated in NaOH solution to remove impurities, such as pectin, waxy and fatty substances and increase the extent of cellulose swelling. KF samples were soaked in a solution of 8 g/L NaOH and 1 g/L wetting agent using a liquor ratio of 1:50 (the ratio of fiber weight to solution volume) at 90 °C for 2 h, after which they were washed in deionized water until the pH of the washing solution was around 7, and finally dried in an oven at 70 °C.

Alkalized KF was used for phosphorylation. The phosphorylation was divided into three steps: (a) KF samples were soaked in an aqueous solution of PA and urea using a bath ratio of 1:50 at 80 °C for 1 h, and then the squeezed KF samples were dried in an oven at 70 °C, (b) the phosphorylation reaction of dried KF samples was carried out in an oven at high temperatures ranging from 120 to 160 °C for 30 to 120 min, (c) the reaction products were washed sequentially in water and in a solution of 2 g/L soap chip using a bath ratio of 1:50 at 40 °C for 30 min, rinsed in water and dried.

In order to investigate the optimum process for the phosphorylation of KF, single-factor experiments were carried out in terms of the amount of PA, the amount of urea, the reaction temperature, and the reaction time. In these experiments, 1 g of alkalized KF was used. For the dependence of PA amount, 2–10 g of PA, 6 g of urea, 150 °C, and 90 min were used. For the dependence of urea amount, 6 g of PA, 2–10 g of urea, 150 °C, and 90 min were used. For the dependence of temperature, 6 g of PA, 6 g of urea, 120–160 °C, and 90 min were used. For the dependence of time, 6 g of PA, 6 g of urea, 150 °C, and 30–120 min were used.

For the instrumental analyses, KF samples were phosphorylated using 2, 4, and 6 g of PA together with 6 g of urea at 150 °C for 90 min, which were labeled as KFPA-2, KFPA-4, and KFPA-6, respectively.

### 3.3. Characterizations and Measurements

The weight increase percentage of phosphorylated KF was used to express the degree of phosphorylation, and it was calculated by the following formula:

(1) $$\mathrm{Weight}\;\mathrm{increase}\;(\%)=100\times \frac{W_{1}-W_{0}}{W_{0}}$$

where *W*_1_ and *W*_0_ refer to the weight of KF samples before and after phosphorylation, respectively. These samples were weighed after being completely dried at 70 °C.

The phosphorus content of phosphorylated KF was measured by ICP-AES on ICAP 6300 DUO (Thermo Fisher Scientific Inc., Waltham, MA, USA), and the argon plasma wavelength was 178.284 nm.

The FT-IR spectroscopy was measured by Nicolet 5700 FT-IR spectrometer (Thermo Fisher Scientific Inc., Waltham, MA, USA). All KF powder samples were ground with KBr and pressed into thin sheets before testing. The IR data were collected from 32 scans with a resolution of 4.0 cm^−1^.

The ^31^P solid-state NMR spectrum of phosphorylated KF was recorded with AvanceIII-400 MHz solid-state NMR spectrometer (Bruker Co., Fällanden, Switzerland).

The thermal stability of KF samples was studied by the TG analysis. TG curves were recorded using Diamond 5700 thermal analyzer (Perkin-Elmer Inc., Waltham, MA, USA) at a gas flow (10 mL/min) and a heating rate of 10 °C/min. The initial weight of each sample was controlled between 5 and 10 mg.

The heat release performance of KF samples was measured by FTT 0001 pyrolysis combustion flow calorimeter (Fire Testing Technology Ltd., East Grinstead, UK).The samples were heated at 1 °C/s.

In order to evaluate the flammability, KF samples were first placed in a cylindrical mold with an appropriate amount of deionized water and then shaped at 110 °C in a hot stamping machine; the resulting sheet samples were burned in YG815B automatic vertical combustion cabinet (Ningbo Textile Instrument Factory, Ningbo, China).

The topographies of raw KF and phosphorylated KF samples, as well as their burned residues produced in the flammability experiment, were observed by Hitachi S-4800 and Regulus 8100 SEM (Hitachi High Technologies America, Inc., Schaumburg, IL, USA).

The Raman spectroscopy was measured by XploRA plus (HORIBA Jobin Yvon S.A.S., Longjumeau Cedex, France) to analyze the graphitization of char residues.

## 4. Conclusions

In this study, the optimal conditions of the phosphorylation of KFusing PA were discussed, and the thermal stability, pyrolysis combustion, flammability, and charring performances of phosphorylated KF were investigated. The optimal phosphorylation reaction conditions were found to be 6 g of PA, 6 g of urea, 150 °C, and 90 min. The phosphorus content of phosphorylated KF prepared using 6 g of PA was 6.23% and still reached 5.65% even after five cycles of soaping, presenting good washing resistance. The phosphorylation reaction of KF with PA was demonstrated by the FT-IR and ^31^PNMR spectroscopic analyses, which reveal the formation of phosphate ester bonds between KF and PA. The TG and PCFC analyses demonstrated that phosphorylated KF was more sensitive to thermal degradation at low temperatures than raw KF. During the pyrolysis combustion, phosphorylated KF exhibited low heat release capability. In the vertical combustion test, phosphorylated KF was very difficult to ignite, presenting excellent flame retardancy. The TG and PCFC analyses of phosphorylated KF and the morphological study of burned phosphorylated KF revealed that PA imparted good charring ability to KF. The Raman spectra illustrated that the char residue of phosphorylated KF was more graphitized than that of raw KF. Therefore, phosphorylated KF is an excellent flame-retardant material.

## Figures and Tables

**Table 1:** TG data of KF and phosphorylated KF samples prepared at various PA dosages (PA 2, 4, and 6 g, 150 °C, and 90 min).

Sample	The First Weight Loss					The Second Weight Loss	
	*T*_o_ (°C)	*T*_e_ (°C)	*T*_max_ (°C)	Residue at *T*_max_ (%)	Residue at *T*_e_ (%)	Residue at 500 °C (%)	Residue at 700 °C (%)
In N_2_							
KF	282	359	344.6	40.7	30.7	16.7	8.3
KFPA-2	213	287	277.3	64.5	56.2	39.8	30.2
KFPA-4	212	271	266.5	65.9	61.1	46.6	36.4
KFPA-6	218	270	267.1	68.3	63.7	49.1	42.6
In air							
KF	276	330	319.5	40.2	28.5	0.4	0.4
KFPA-2	220	276	270.8	64.3	57.0	24.5	4.6
KFPA-4	210	267	262.6	67.9	62.2	33.3	3.7
KFPA-6	214	263	260.5	66.6	62.2	32.2	3.3

Note: *T*_o_ is the onset decomposition temperature, *T*_e_ is the end decomposition temperature, and *T*_max_ is the maximum decomposition rate temperature.

## Data Availability

The data are available upon request.

## Abbreviations

The following abbreviations are used in this manuscript:

DTG	Differential thermogravimetric
FT-IR	Fourier transform infrared
KF	Kapok fiber
NMR	Nuclear magnetic resonance
HRC	Heat release capacity
HRR	Heat release rate
ICP-AES	Inductively coupled plasma-atomic emission spectrometry
PA	Phytic acid
PCFC	Pyrolysis combustion flow calorimetry
pHRR	Peak heat release rate
SEM	Scanning electron microscope
TG	Thermogravimetric
THR	Total heat release
